# A Missing Link in Radial Ion Transport: Ion Transporters in the Endodermis

**DOI:** 10.3389/fpls.2019.00713

**Published:** 2019-06-04

**Authors:** Zhulatai Bao, Juan Bai, Hongchang Cui, Chunmei Gong

**Affiliations:** ^1^College of Life Sciences, Northwest A&F University, Yangling, China; ^2^College of Horticulture, Northwest A&F University, Yangling, China; ^3^Department of Biological Science, Florida State University, Tallahassee, FL, United States

**Keywords:** root, endodermis, Casparian strip, suberin, ion transporter

## Abstract

In higher plants, roots have important functions, such as the acquisition of water and ions, as well as transportation into the aerial parts of the plant via the xylem vessels. Radial ion transport in the root is strongly regulated in the endodermis, which is characterized by the presence of the Casparian strip (CS) and suberin lamellae. Although tremendous progress has been made with regard to the ion transporters and endodermal cells, little is known about the relationship between the ion transporters in the endodermis and ion homeostasis in aboveground tissues. This review summarizes the current knowledge about the location of the ion transporters (or channels) in the endodermis. We mainly discuss the effects of mutants related to the CS and/or suberin formation on the role of endodermal ion transporters in ion homeostasis. We also wish to emphasize the critical role of endodermal ion transporters in the regulation of radial ion transport in the root.

## Introduction

The root system plays an indispensable role during the uptake of mineral ions. In the roots, various cell layers are organized in concentric rings around the vascular tissues, which are, from the outside to the inside, the epidermis, exodermis (if exists), cortex, and endodermis (Steudle and Peterson, 1998). To reach the xylem, however, ions traveling in the apoplast have to pass through the endodermis via transporter proteins, due to the presence of the Casparian strip (CS) (Pauluzzi et al., 2012; Andersen et al., 2015). Therefore, ion transporters located in the plasma membrane of the endodermis are particularly important and deserve more attention.

**FIGURE 1:**
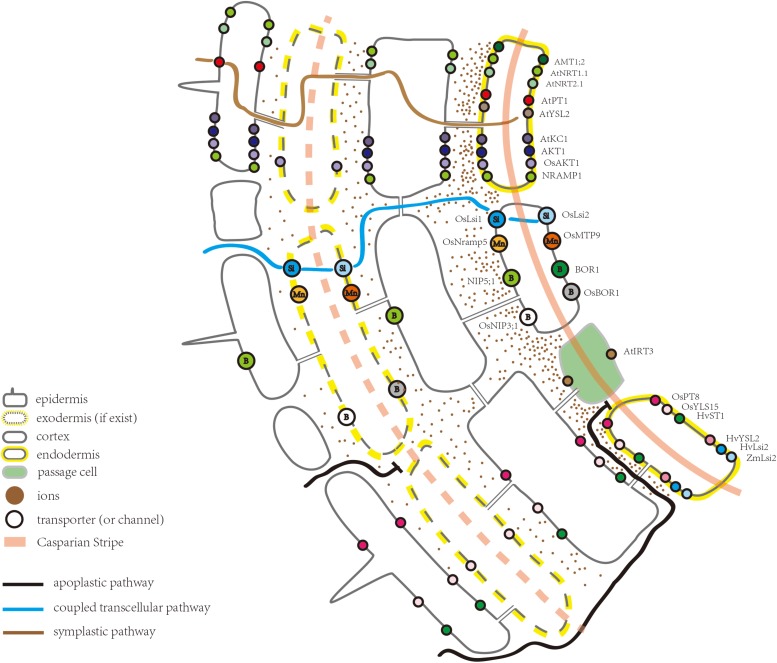
Schematic of part of a root highlighting the ion transporters (or channels) located in the endodermis.

Substantial progress has been made in the identification of various types of ion transporters in the root, in addition to the understanding of ion absorption and long-distance transport (Shi et al., 2002; Lin et al., 2008; Véry et al., 2014). Many transporter proteins in *Arabidopsis thaliana* have also been uncovered by membrane proteomic approaches (Alexandersson et al., 2004; Brugiere et al., 2004; Carter et al., 2004). Among the membrane ion transporters, a few are expressed ubiquitously while others are located only in the endodermis. From a functional point of view, ion transporters expressed in the endodermis represent a missing link in our understanding of the root-to-shoot ion transport pathway, which is essential to ion homeostasis in aboveground tissues. The endodermal ion transporters (or channels) discovered in *Arabidopsis thaliana*, rice (*Oryza sativa*), barley (*Hordeum vulgare*), and maize (*Zea mays*) are summarized in Table 1 and Figure 1.

**TABLE 1:** Localization of ion transporter (or channel) genes on the root cell.

**Gene**	**Substrate**	**ep**	**ex**	**co**	**en**	**Polarity**	**References**
*AtNRT1.1*	Nitrate	√	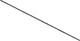	√	√		Huang et al.,1996
*AtNRT2.1*	Nitrate	√	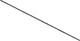	√	√		Nazoa et al.,2003
*AMT1;2*	Ammonium		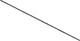		√		Neuhäuser et al.,2007
*AtPT1*	Phosphate	√	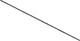		√		Karthikeyan et al.,2002
*AKT1*	Potasium	√	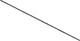	√	√		Lagarde et al.,1996
*AtKC1*	Potasium	√	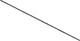	√	√		Reintanz et al.,2002
*AtYSL2*	Iron/Zinc		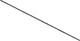		√		Schaaf et al.,2005
*AtIRT3*	Iron/Zinc		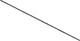		√		Lin et al.,2009
*NIP5;1*	Boron	√	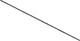	√	√	Distal	Takano et al.,2008
*BOR1*	Boron		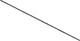		√	Proximal	Takano et al.,2008
*Nramp1*	Manganese	√	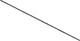	√	√		Cailliatte et al.,2010
*HvYSL2*	Iron		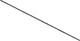		√		Araki et al.,2011
*HvST1*	Sulfate	√	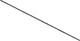	√	√		Rae and Smith,2002
*HvLsi2*	Silicon		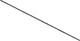		√		Mitani et al.,2009
*ZmLsi2*	Silicon				√		Mitani et al.,2009
*OsPT8*	Phosphate	√		√	√		Jia et al.,2011
*OsAKT1*	Potasium	√	√	√	√		Li et al.,2014
*OsYLS15*	Iron	√	√	√	√		Lee et al.,2009
*OsLsi1*	Silicon		√		√	Distal	Ma et al.,2006
*OsLsi2*	Silicon		√		√	Proximal	Ma and Yamaji,2015
*OsNramp5*	Manganese/Cadmium		√		√	Distal	Sasaki et al.,2012
*OsMTP9*	Manganese		√		√	Proximal	Ueno et al.,2015
*OsNIP3;1*	Boron		√		√	Distal	Takano et al.,2008
*OsBOR1*	Boron		√		√	Proximal	Nakagawa et al.,2007

^*^Slash represents the species lacking exodermal cell. ep, Epidermis; ex, exodermis; co, cortex; en, endodermis.

## Transport Routes for Ions in the Root

The movement of water and solutes in the root has been classified into three pathways: the apoplastic pathway, symplastic pathway, and coupled transcellular pathways (Figure 1; Steudle, 2000). The apoplastic pathway provides a route for ions to reach the stele, by diffusing through the cell walls and free spaces between cells (Barberon and Geldner, 2014). The rate of diffusion depends upon the ionic gradient between the external solution and apoplastic free space (Barberon and Geldner, 2014). A strictly apoplastic flow from the soil to the stele can occur only prior to CS differentiation or at the site of CS disruption, for example, at a site of lateral root emergence (Krishnamurthy et al., 2011; Barberon and Geldner, 2014). Apoplastic flow is less controlled and non-selective where molecules diffuse along the concentration gradient or move with the bulk water flow driven by transpiration (Steudle and Peterson, 1998; Steudle, 2000; Enstone et al., 2003; Barberon and Geldner, 2014). After uptake by the epidermal cells, ions can be transported via the symplastic pathway through the connections between the cells provided by plasmodesmata (Andersen et al., 2015). The transport rate through the symplastic pathway depends upon the density and size exclusion limit of plasmodesmata (Stahl and Simon, 2013; Barberon and Geldner, 2014). Additionally, ions can repeatedly cross plasma membranes through transporter proteins, a transport route usually referred to as the coupled transcellular pathway (Robbins et al., 2014). Overall, ion transportation via polarly localized ion transporters in the plasma membrane is more directional compared to the apoplastic pathway (Geldner, 2013).

## CS and Suberin Make the Endodermis an Effective Barrier

In *Arabidopsis*, endodermis differentiation can be divided into two stages: the CS formation at the first stage, and “patchy” appearance of suberin lamellae at the second stage that gradually enclose the whole cell in an upward direction along the longitudinal axis (Naseer et al., 2012; Geldner, 2013; Barberon and Geldner, 2014; Andersen et al., 2015; Barberon, 2016). The CS is a belt-like lignin-based structure, which forms in the middle of the anticlinal walls of the endodermal cells (Enstone et al., 2003; Andersen et al., 2015). The CS is tightly adhered to the plasma membrane and spans the apoplastic space between adjacent endodermal cells, thus completely isolating the apoplast of the stele from the apoplast of the outer tissue layers (Naseer et al., 2012; Robbins et al., 2014). Although semi-permissive, the plasma membrane sometimes fails to prevent the entry of undesirable compounds due to passive diffusion (Robbins et al., 2014). Deposited as a secondary cell wall between the primary cell wall and plasma membrane, the suberin lamellae can cover the entire surface of the endodermal cells as the root matures (Geldner, 2013). The presence of suberin lamellae potentially blocks the direct uptake of nutrients from the apoplast into the endodermal cells (Geldner, 2013; Robbins et al., 2014). Ultimately, the efficient and selective sorting of nutrients is accomplished at the endodermis level (Enstone et al., 2003; Alassimone et al., 2012). For example, in rice, exposure to moderate salt stress results in the enhancement of suberization and significant reduction in sodium accumulation in the shoot (Krishnamurthy et al., 2009, 2011). In maize roots, accelerating suberin deposition in the endodermis has been shown to restrict the radial apoplastic movement of cadmium (Cd) and Cd loading in the xylem (Lux et al., 2011).

Together with previous studies, Barberon et al. (2016) clearly defined the function of the CS from that of the suberin lamellae: the CS blocks the passage between the endodermal cells, while suberin lamellae suppresses the uptake into the cytoplasm of the endodermis (Naseer et al., 2012; Hosmani et al., 2013; Pfister et al., 2014; Kamiya et al., 2015; Barberon et al., 2016). In *Arabidopsis*, ion accumulation in the leaves of CS defective mutants, such as *myb36*, *esb1*, *casp1casp3*, and *sgn3*, is significantly altered under normal growth conditions (Baxter et al., 2009; Roppolo et al., 2011; Hosmani et al., 2013; Pfister et al., 2014; Kamiya et al., 2015; Barberon et al., 2016). Among them, *myb36*, *esb1*, and *casp1casp3* show compensatory deposition of suberin (Pfister et al., 2014). The potassium (K) content in a *sgn3* leaf is decreased, which is accompanied by an increase in magnesium (Mg) (Pfister et al., 2014). However, the ion concentrations in *myb36*, *esb1*, and *casp1casp3* leaves have been further disrupted due to suberin enhancement, which suggests that suberin deposition impacts the ion transport from the roots to shoots. As a secondary cell wall modification, suberin restricts the access of nutrients to the plasma membrane of the endodermis (Geldner, 2013). Hence, it can also be suggested that suberin hinders the access of ions to endodermal ion transporters. Concerning leaf ion homeostasis, striking similarities have been reported between *myb36*, *esb1*, and *casp1casp3*, all of which show a reduction in the iron (Fe) and manganese (Mn) contents (Hosmani et al., 2013; Kamiya et al., 2015). Notably, the Fe transporter, AtYSL2, is located in the endodermis (Figure 1; Schaaf et al., 2005). In rice, the natural resistance-associated macrophage protein 5 (OsNramp5) and metal tolerance protein 9 (OsMTP9) are known to be located in the distal and proximal sides of the endodermis, respectively, mediating the direct transport of Mn across the endodermal cells (Figure 1 and Table 1; Sasaki et al., 2012; Ueno et al., 2015; Shao et al., 2017). The spatial distribution and cellular localization of Mn transporters in *Arabidopsis* are not as well understood as those in rice (Shao et al., 2017). However, it is known that in *Arabidopsis*, Fe- and Mn-deficiency decreases suberization, compared to control plants. A dramatic reduction in the extent of suberization has been observed in mutants of the *iron-regulated transporter1* (*irt1*) and *natural-resistance-associated macrophage proteins1* (*nramp1*), consistent with the effects of Fe and Mn deficiency on suberization (Barberon et al., 2016). One possible explanation for the alteration in the Fe and Mn contents in *myb36*, *esb1*, and *casp1casp3* is that the enhanced suberization in these mutants impedes the transporter protein mediated Fe/Mn transport into the endodermis. Moreover, suberization of both the endodermis and exodermis in rice is delayed under ${\text{NH}}_{4}^{+}$-deficient conditions (Ranathunge et al., 2016). In contrast, suberin is deposited early and close to the apex of the roots under high ${\text{NH}}_{4}^{+}$ conditions (Ranathunge et al., 2016). The authors also suggest that, compared to a fully suberized cell layer, patchy suberin may provide the transporters in the plasma membrane a higher access to ${\text{NH}}_{4}^{+}$ (Ranathunge et al., 2016). In a recent study, it was shown that *esb1* and *myb36* had a lower amount of ${}^{15}{\text{NH}}_{4}^{+}$ in the shoots (Duan et al., 2018). Therefore, a detailed analysis about the impacts of suberin deposition on endodermal ion transporters is required.

Magnesium is another element that is affected in all the above-described mutants. Magnesium transport 1 (MGT1) and MGT6 are two plasma membrane transporters involved in the uptake of Mg in *Arabidopsis* roots (Li et al., 2001; Mao et al., 2014). Although both of them are expressed in the vascular tissues, MGT6 is significantly up-regulated when seedlings are transferred from Mg-sufficient to -deficient conditions (Mao et al., 2014). Interestingly, the expression domain of MGT6 is even extended to the cortex and epidermis (Mao et al., 2014). Additionally, Ogura et al. (2018) demonstrated that Mg uptake under Mg-sufficient conditions is particularly sensitive to the inhibitory effects of gadolinium (Gd) and Fe, but not of K. Nevertheless, the information available about the effects of Mg-deficiency on suberin deposition is still lacking.

The research described above lends strong support to the notion that the endodermis is a checkpoint for ion accumulation in the shoots.

## Ion Transporters in the Endodermis

### Non-polarly Localized Transporters

As an important component of the endodermal barrier, ion transporters determine which ion can enter the stele and, thus, act as a gateway controlling the ins and outs of ions. Figure 1 illustrates the ion transporters (or channels) with an endodermal localization, including those for nitrate, ammonium, phosphate, potassium, sulfate, iron, and zinc. Except for iron transporter AtIRT3, however, these transporters (or channels) are not exclusively expressed in the endodermis (Lin et al., 2009). Through these transporters (or channels), ions can enter the symplastic route for radial transportation toward the root stele and are eventually loaded into the xylem. In *Arabidopsis* roots, the nitrate transporter2.1 (AtNRT2.1), ammonium transporter1;2 (AMT1;2), chlorate resistant 1 (CHL1/AtNRT1.1), and inward-rectifier K^+^ channel (AKT1) are all highly expressed in the mature region of the root. However, the degree of suberization in the mature region remains unclear. Huang et al. (1996) suggested that CHL1 and AKT1 have similar but unidentical patterns of gene expression. In root cells close to the root tip, *CHL1* is mainly expressed in the epidermal cells, whereas in the older part, *CHL1* is expressed in the cortex and endodermis, and its expression domain becomes restricted to the endodermis (Huang et al., 1996). Thus, it seems the cells that are important for ion uptake, tend to be more internally localized as the roots become more mature (Huang et al., 1996). This trend is consistent with and probably due to a more conductive xylem tissue in the mature zone and a higher water demand, driven by the transpiration stream, where water demand is essentially driven by cell elongation (Hachez et al., 2006). In any case, there is no doubt that transporters expressed in the endodermis play an important role in controlling the uptake of ions from the apoplast of the outer cell layers into the endodermal cytoplasm and further entry into the stele. In line with this notion, some studies have suggested that the endodermis serves as a filter, damping the ion movement at the site of the CS and, as a result, ion concentrations are likely higher near the endodermis (Enstone et al., 2003). In support of this view, in *Arabidopsis* roots, ammonium is confined to the apoplast closest to the endodermis, and AMT1;2 has a much higher Km than that of the transporters located in the rhizodermis, such as AMT1;1 and AMT1;3 (Yuan et al., 2007). This observation has lead (Yuan et al., 2007) to conclude that there is a spatial distribution of AMT1 transporters – those possessing higher substrate affinities are located in the outer root cells, whereas those with a lower affinity are located at the end of the apoplastic transport pathway. Under high ammonium concentrations, AMT1;2 in the endodermis favors ammonium partitioning to the shoot (Duan et al., 2018). In addition, the expression of another ammonium transporter, *AMT2;1*, becomes more confined to the endodermal and pericycle cells when only ammonium is present (Giehl et al., 2017). As a result, the co-expression of AMT2;1 with AMT1;2 in the *amt1;1 amt1;2 amt1;3 amt2;1* quadruple mutant, significantly enhances the ^15^N translocation to the shoot (Giehl et al., 2017). These studies indicate that the endodermal localization of ammonium transporters is critical for ammonium partitioning to the shoot.

### Polarly Localized Transporters

The asymmetric distribution of the influx and efflux proteins in endodermal cells is believed to be a key factor driving the directional solute movement toward the stele and preventing backflow when transpiration is low (Sakurai et al., 2008; Robbins et al., 2014). However, only a few examples of the polar localization of ion transporters (or channels) have been reported (Figure 1). For example, Ma and Yamaji (2015) demonstrated that polar localization of the silicon influx channel, low silicon rice 1, and silicon exporter, silicon rice2, are essential for silicon transport in rice roots (Ma et al., 2006, 2007; Ma and Yamaji, 2015). Both of them are expressed in the exodermis and endodermis, providing the coupled transcellular pathway directing Si toward the stele. Like the Si transporter, the boric acid/borate exporter (OsBOR1) in rice spans the exodermal CS, as well as the endodermis. Together, these OsBOR1 proteins provide a transcellular efflux pathway for the directional movement of boron (B) (Nakagawa et al., 2007). In *Arabidopsis*, the boric acid channel, NIP5;1, is required for B uptake under B deficiency conditions, whilst the boric acid exporter, BOR1, excludes borate from cells close to the xylem (Figure 1; Takano et al., 2005, 2008). Wang et al. (2017) demonstrated that the polar localization of NIP5;1 is maintained by clathrin-mediated endocytosis (Nakamura and Grebe, 2018). However, high levels of B are toxic to plants. To avoid toxicity, BOR1 is transported to the vacuole wherein it is degraded (Takano et al., 2005, 2010; Nakamura and Grebe, 2018). Another mechanism to reduce BOR1 expression under high B levels is through the transcriptional repression of BOR1, mediated by BOR1 5′-UTR (Aibara et al., 2018). In rice, OsNramp5 is located at the distal side of the exodermis and endodermis and is responsible for the transportation of Cd (not essential for plant growth), Mn, and Fe (Figure 1; Sasaki et al., 2012). However, the *Arabidopsis Nramp1* is located in all root cell layers (Figure 1; Cailliatte et al., 2010). When knocked out, *Nramp1* and *OsNramp5* mutants also have different phenotypes (Sasaki et al., 2012). In contrast to the *OsNramp5* knockout line, the decreased Mn concentration in the shoot of *Nramp1* knockout lines can be rescued by a high Mn concentration (Sasaki et al., 2012). This result also suggests that the mediated transport of Mn is indispensable for the normal growth and development of rice (Sasaki et al., 2012). The uptake systems for Si, B, and Mn in rice roots have been summarized by Sasaki et al. (2016) and Che et al. (2018).

The contribution of endodermal ion transporters to the ion accumulation in aboveground tissues remains unclear. To answer this question, the tissue specific knockdown of certain ion transporters in the endodermis is required. To our knowledge, only a few studies have demonstrated that Cd accumulation in rice is strongly decreased when the function of OsNramp5 is defective (Clemens et al., 2012; Sasaki et al., 2012). Sasaki et al. (2012) also demonstrated that the knockout of OsNramp5 results in almost a complete loss of the ability to uptake Cd. Research on *Noccaea caerulescens*, a Zn/Cd hyperaccumulator, revealed that the endodermal-expressed *NcNramp1* gene plays a key role in Cd translocation into the stele (Milner et al., 2014).

## Conclusion and Future Perspectives

The importance of ion transporters in plant growth and development is beyond the question. Their positioning in the endodermis makes them a crucial player in the regulation of ion transport from the roots to shoots. To compare ion transportation across endodermal cells with other cell layers, the ion flux of different cell layers needs to be measured. However, it is not yet possible to directly measure the ion flux of the inner cell layers of the root. This problem could be resolved in the near future, for example, by the non-invasive microelectrode ion flux estimation (MIFE) technique, which has been used to measure the net fluxes of different ions within the root surface. Another issue yet to be addressed is the contribution of endodermal ion transporters to radial ion transport from the roots to shoots. Many ion transporters, such as CHL1, AMT1.1, and AKT1, can serve as sensors of nitrate, ammonium, and potassium, respectively (Ho et al., 2009; Ho and Tsay, 2010). Accordingly, it would be interesting to study whether endodermal ion transporters play such a role, thus making the endodermis a signaling hub. According to Dinneny (2014), the endodermis acts as a signaling center where multiple hormone signals, such as auxin, gibberellic acid, abscisic acid, and strigolactones, are integrated. Data from Li S. et al. (2016) indicate that the ion transporter Nramp1 is co-expressed with lincRNAs. Considering that lincRNAs play important roles during development and in response to stress, they are deserving further research.

As the gene expression atlas at a single cell resolution has become available, cell type specific ion transporters have been identified (Gifford et al., 2008; Geng et al., 2013; Li B. et al., 2016; Ryu et al., 2019). For instance, through the reanalysis of the published database of Evrard et al. (2012), Evrard (2013) and Li B. et al. (2016) identified chloride transporters localized in the stele. Similarly, ion transporters preferentially expressed in the endodermis have also been reported, such as AT4G02090 and AT5G14880, which encode the multidrug resistance protein ABC transporter family protein and potassium transporter family protein, respectively (Geng et al., 2013; Ryu et al., 2019).

The formation of the CS and suberin lamellae is closely correlated with the leaf ionome in the *esb1*, *casp1casp3*, *myb36*, and *sgn3* mutants (Baxter et al., 2009; Roppolo et al., 2011; Hosmani et al., 2013; Pfister et al., 2014; Kamiya et al., 2015; Barberon et al., 2016). More importantly, the plasticity of suberization in response to nutritional stress has already been highlighted (Barberon et al., 2016). Nevertheless, a number of questions still remain concerning the function of ion transporters in the context of the endodermis. If the degree of suberization changes in the endodermis, will the expression of ion transporters be affected? For those located within the endodermal cell, will their location change? Cell type level investigation using the fluorescence-activated cell sorting (FACS) technique combined with a time series of transcriptomic analyses, as described recently in Walker et al. (2017), may aid in answering these questions.

## Author Contributions

ZB drafted and revised the manuscript. HC, CG, and JB revised the manuscript.

## Conflict of Interest Statement

The authors declare that the research was conducted in the absence of any commercial or financial relationships that could be construed as a potential conflict of interest.
